# Expansion of the medical cannabis market in Brazil and regulatory challenges

**DOI:** 10.1590/0102-311XEN088624

**Published:** 2024-12-20

**Authors:** Cláudia Du Bocage Santos Pinto, Ângela Esher, Cátia Verônica dos Santos Oliveira, Claudia Garcia Serpa Osorio-de-Castro

**Affiliations:** 1 Faculdade de Medicina, Universidade Federal de Mato Grosso do Sul, Campo Grande, Brasil.; 2 Escola Nacional de Saúde Pública Sergio Arouca, Fundação Oswaldo Cruz, Rio de Janeiro, Brasil.

## Growing demand for cannabis-based products

Brazil spent around BRL 165 million with the public supply of cannabis-derived products from 2015 to mid-2023, with about half of this amount resulting from lawsuits filed against the government ^1^. A Federal Bill ^2^ is currently being analyzed in Brazil’s Chamber of Deputies to establish the National Policy for the Supply of Cannabis-Derived Products by the Brazilian Unified National Health System (SUS, acronym in Portuguese). Several bill have already been passed or are under analysis in several states and municipalities nationwide ^3^.

The growing interest in these products is also the result of the work of several entities, which focus on the growth and artisanal manufacture of cannabis-derived products for medicinal purposes ^4^. The scenario also shows the emergence of industries in Brazil, which have sanitary authorization to manufacture and sell, as well as importers that facilitate the entry of numerous products authorized for import, coming from several countries such as the United States, the United Kingdom, Switzerland, the Netherlands, and Uruguay ^5^ ^,^ ^6^.

The established demand is encouraged by advertising campaigns announcing the therapeutic benefits of cannabis-derived products and promoting their alleged and diverse medicinal properties, most of which still lack scientific evidence to support their use by the population. This supply contributes to the understanding that any product containing cannabis should be considered a medicine; however, what has been seen is the idea of products and cannabis itself as a panacea ^7^.

## Regulatory framework and evidence for cannabis use

In Brazil, there is only one cannabis derived product classified as a medicine. The medical use of these products is a controversial issue, as *Cannabis sativa* is considered a prohibited plant according to Brazilian Minstry of Health *Ordinance n. 344/1998* (list E) ^8^. However, since cannabidiol (CBD) started being used to treat refractory epilepsy in children, there has been an increase in demand for its medical use in Brazil ^9^. Brazilian Health Regulatory Agency (Anvisa, acronym in Portuguese) included CBD in the C1 list, the same classification as other substances under special control ^10^.

In this context, regulations for the medical use of cannabis have been developed in the country. Resolutions have authorized the import of cannabis-containing products, provided that their producers or distributors are authorized by the health authorities of their countries of origin ^11^ ^,^ ^12^. Currently, *Resolution n. 660* establishes the criteria and procedures for import by individuals for personal use only, and with a prescription from a healthcare professional ^6^.

Anvisa has established requirements for marketing, prescribing, dispensing, monitoring, and inspection of products authorized for manufacture in Brazil, which can only be sold in ready-made form (compounding not authorized) and with a medical prescription. Products with up to 0.2% THC require a “B”-type prescription, and those with a higher content for palliative care should have an “A”-type prescription ^5^.

On the other hand, the legal establishment authorized patient associations to grow, compound, prepare, produce, store, transport, dispense, and research *C. sativa* ^13^; and compounding pharmacies were granted the right to make plant-derived preparations. According to the Judiciary, this would imply a trade restriction for foreign companies to manufacture cannabis-based products ^14^. The arguments of bodies that are not technically prepared to act in health regulation divert the attention from the issues that need to be urgently resolved by Anvisa.

Today, the regulatory scenario for medical cannabis in Brazil has three categories: (i) Anvisa market-approved medicines, with only one example, a product containing synthetic cannabinoids; (ii) products authorized for manufacturing and sold in pharmacies, based on pure cannabidiol or cannabis extract, for which companies are only required to have a certificate of good manufacturing practices ^5^. Around 30 products have a valid license ^15^; and (iii) products with an import license that present a variety of cannabinoid compositions (full spectrum, broad spectrum, and isolate cannabinoid oils), in addition to different dosages forms (capsules, sublingual aerosols, patches, lotions, creams, and ointments), and concentrations ^16^. From 2015 to 2024, Anvisa published and updated about 30 lists of products with import authorization. The first list had 11 products ^11^, and the most recent list, 582 ^16^. From 2015 to mid-2023, around 270,000 import authorizations were granted by Anvisa, allowing patients to obtain such products ^1^.

Growing demand and inadequate regulation are complemented by the low quality of evidence for most potential indications for using cannabis-based products. The effects of synthetic or natural cannabinoids on chronic pain, spasticity, nausea/vomiting, loss of appetite, amyotrophic lateral sclerosis, irritable bowel syndrome, multiple sclerosis, Huntington’s chorea, epilepsy, dystonia, Parkinson’s disease, glaucoma, attention deficit hyperactivity disorder, anorexia nervosa, anxiety, autism, dementia, depression, schizophrenia, post-traumatic stress, sleep disorders, drug addiction, Tourette’s syndrome, among others, have been studied in recent years. However, only a few indications have a high or moderate level of evidence ^17^.

For cannabidiol, the level of evidence was high for epilepsy and moderate for Parkinson’s disease. Moderate evidence was found for nabiximol for chronic pain, spasticity, sleep, depression, and drug addiction; and moderate evidence was observed for dronabinol for chronic pain, loss of appetite, and Tourette’s syndrome ^17^. Studies are ongoing for other indications and the available evidence is insufficient ^18^ ^,^ ^19^.

## The issue of safety

According to the Brazilian health legislation, no product for medical use can be manufactured, sold or delivered for consumption until it has been registered ^20^. Despite the creation of a specific category, these products should comply with health regulations. After a post-approval 5-year period, products authorized for manufacture and sale must be registered with Anvisa. Until then, manufacturers, prescribers, and patients are responsible for the risks - in this case, the patients must sign an informed consent form. This creates a context of possible information asymmetry that could lead to a higher burden for patients.

But what about the number of products authorized for import? There is no registration planned for these products. Will the almost 600 products in this category continue to rely on fragile or even non-existent regulatory requirements that are currently in force? This situation favors individuals who obtain products through their own resources or through the courts. Litigation for access to products, fueled by social pressure, is made tense due to the relative lack of evidence and legal uncertainty, given that these products are not medicines. The actions of CONITEC (the Brazilian National Commission for the Incorporation of Technology into the SUS) are essential to address this issue in favor of the sustainability of the SUS.

The therapeutic use of an active ingredient depends on proof of efficacy and safety for a given indication. Adverse events of medical cannabis, as well as evidence of efficacy, have not yet presented conclusive results. A moderate association has been observed for the risk of adverse events with nabilone, nabiximol and CBD ^17^. Cardiovascular, psychiatric, gastrointestinal and immune system events have been reported in the literature ^21^. With THC-containing products, the patient may suffer disorders such as psychosis, related either to THC concentration or to chronic use, or addiction-like disorders, which are more severe in susceptible patients ^19^ ^,^ ^21^ ^,^ ^22^ ^,^ ^23^.

In the United States, in the debate on the regulation of medical cannabis, the issue of safety has focused on limited information on adverse effects of approved products, indicating the importance of conducting clinical trials ^24^.

In Brazil, monitoring of adverse events requires Anvisa to encourage prescribers and patients to report such events, with the coordination of professional councils to protect patients and ensure evidence-based indications.

## Who shapes regulation?

Although contributions from society help shape regulation, regulatory gaps pave the way for a market that is not always aligned with the interests of health. The result is a gradual increase in the number of companies seeking to operate in this niche, which is both lucrative and permeable.

Anvisa creates alternatives to make cannabis-containing products, which are not medicines and, therefore, have not been evaluated regarding efficacy, safety or quality, available to patients by means of a prescription only. In this case, the provision by the SUS of products that do not meet or do not comply with the health standards established in the legislation is even more serious.

Anvisa is expected to take a more assertive position regarding the regulation of cannabis for the indicated uses according to the evidence and the availability of products authorized for import and sale in the country. Anvisa has historically shown its determination to protect the Brazilian population; for example, when handling the COVID-19 pandemic. The same position is expected in the regulation of the cannabis market, as well as other relevant markets that affect the health of the population.
