# Supplementary material for: Expansion of the medical cannabis market in Brazil and regulatory challenges
Source: Cad Saude Publica. 2024 Dec 20;40(11):e00088624. doi: 10.1590/0102-311XEN088624 (PMC11654110; doi:10.1590/0102-311XEN088624)
Supplement: Translation

# Translation: A expansão do mercado da cannabis medicinal no Brasil e os desafios da regulação

**DOI:** 10.1590/0102-311XPT088624

**Authors:** Cláudia Du Bocage Santos Pinto, Ângela Esher, Cátia Verônica dos Santos Oliveira, Claudia Garcia Serpa Osorio-de-Castro

**Affiliations:** 1 Faculdade de Medicina, Universidade Federal de Mato Grosso do Sul, Campo Grande, Brasil.; 2 Escola Nacional de Saúde Pública Sergio Arouca, Fundação Oswaldo Cruz, Rio de Janeiro, Brasil.

## A crescente demanda de produtos à base de cannabis

O Brasil gastou cerca de R$ 165 milhões com fornecimento público de produtos contendo *Cannabis sativa*, de 2015 até meados de 2023, sendo cerca de metade desse valor resultado de demandas judiciais impetradas contra o poder público ^1^. Tramita, no Congresso Nacional, um Projeto de Lei (PL) Federal ^2^ visando instituir a Política Nacional de Fornecimento de Produtos Derivados da Cannabis pelo Sistema Único de Saúde (SUS). Diversos PLs já foram aprovados ou estão em tramitação, em vários estados e municípios pelo país ^3^.

O crescente interesse por esses produtos também é resultado da atuação de diversas associações cujas atividades são centradas no cultivo e na fabricação artesanal de produtos derivados de cannabis com fins medicinais ^4^. O panorama é complementado ainda pelo surgimento de indústrias no Brasil com autorização sanitária para a fabricação e a comercialização, e de importadores que viabilizam a entrada de inúmeros produtos autorizados para importação, originários de diversos países, como os Estados Unidos, o Reino Unido, a Suíça, a Holanda e o Uruguai ^5^ ^,^ ^6^.

A demanda, já estabelecida, é incentivada por campanhas publicitárias anunciando os benefícios terapêuticos de produtos contendo cannabis e propalando suas pretensas e diversas propriedades medicinais, a maior parte das quais ainda sem grau de evidência científica que respalde seu uso pela população. Essa oferta contribui para o entendimento de que qualquer produto contendo cannabis seja considerado um medicamento; ao contrário, o que vem se manifestando é a ideia dos produtos e da própria cannabis como panaceia ^7^.

## Marco regulatório e evidências que dão suporte à utilização

No Brasil, há apenas um produto derivado da cannabis classificado como medicamento. A utilização medicinal desses produtos é uma questão controversa, pois a *C. sativa* é considerada planta proscrita pela *Portaria nº 344*, de 12 de maio de 1998 (lista E), do Ministério da Saúde ^8^. Entretanto, a partir do emprego do canabidiol (CBD) para tratamento de epilepsia refratária em crianças, houve aumento na demanda por seu uso medicinal no Brasil ^9^. A Agência Nacional de Vigilância Sanitária (Anvisa) passou a alocar o CBD na lista C1, mesma classificação de outras substâncias sujeitas a controle especial ^10^.

Nesse contexto, foi sendo “construída” uma regulamentação para o uso medicinal da cannabis no país. Resoluções autorizaram importações de produtos contendo cannabis, desde que seus produtores ou distribuidores fossem regularizados pelas autoridades sanitárias de seus países de origem ^11^ ^,^ ^12^. Atualmente a *Resolução de Diretoria Colegiada nº 660*, da Anvisa ^6^, define os critérios e os procedimentos para a importação, desde que por pessoa física, para uso próprio e mediante prescrição de profissional habilitado.

A Anvisa estabeleceu requisitos de comercialização, prescrição, dispensação, monitoramento e fiscalização para os produtos autorizados a fabricação no Brasil, que só podem ser vendidos prontos (sem manipulação pelas farmácias) e com prescrição médica. Produtos com até 0,2% de tetrahidrocanabinol (THC) demandam notificação de receita “B”, e aqueles com maior teor, destinados a cuidados paliativos, notificação de receita “A” ^5^.

Por outro lado, a justiça autorizou associações de pacientes a cultivar, manipular, preparar, produzir, armazenar, transportar, dispensar e pesquisar com *C. sativa* ^13^; e há farmácias magistrais que obtiveram o direito de manipular a planta. Segundo o entendimento do judiciário, haveria reserva de mercado a empresas estrangeiras no fabrico de produtos à base de cannabis ^14^. Argumentos postulados por instâncias não capacitadas para atuar na regulação sanitária desviam o foco das questões que necessitam de urgente resolução pela Anvisa.

Atualmente, o cenário regulatório da cannabis medicinal no país inclui três categorias: (1) medicamentos com registro junto à Anvisa, no caso, apenas um contendo canabinóide sintético; (2) produtos autorizados para fabricação, e comercializados em farmácias, à base de canabidiol puro ou de extrato de cannabis, para os quais se exige apenas que as empresas possuam um Certificado de Boas Práticas de Fabricação ^5^, há cerca de 30 produtos com autorização válida ^15^; e (3) produtos com autorização de importação que apresentam uma multiplicidade de composições de canabinóides (óleos *full*, *broad spectrum* ou com canabinóides isolados), além de diversas formas farmacêuticas (cápsulas, aerossóis sublinguais, adesivos, loções, cremes e pomadas) e concentrações ^16^. Entre 2015 e 2024, a Anvisa publicou e atualizou cerca de 30 listas de produtos com autorização de importação. A primeira lista incluía 11 produtos ^11^, e a mais recente 582 ^16^. Entre 2015 e meados de 2023, cerca de 270 mil permissões para importação foram concedidas pela Anvisa para que pacientes pudessem obter tais produtos ^1^.

A demanda crescente e a regulação insuficiente são complementadas pela baixa qualidade de evidências para grande parte das possíveis indicações. Os efeitos de canabinóides sintéticos ou naturais para dor crônica, espasticidade, náusea/vômitos, perda de apetite, esclerose lateral amiotrófica, síndrome do colo irritável, esclerose múltipla, coreia de Huntington, epilepsia, distonia, doença de Parkinson, glaucoma, transtorno do déficit de atenção com hiperatividade, anorexia nervosa, ansiedade, autismo, demência, depressão, esquizofrenia, estresse pós-traumático, distúrbios do sono, drogadição, síndrome de Tourette, entre outros, têm sido investigados nos últimos anos. No entanto, poucas indicações têm evidência de nível alto ou moderado ^17^.

Para o canabidiol, evidências foram altas para epilepsia e moderadas para doença de Parkinson. Evidência moderada foi demonstrada para nabiximol em dor crônica, espasticidade, sono, depressão e drogadição; evidência moderada foi observada para dronabinol em dor crônica, perda de apetite, e síndrome de Tourette ^17^. Para outras indicações, os estudos ainda estão em curso e as evidências disponíveis são fracas ^18^ ^,^ ^19^.

## O problema da segurança

Segundo a legislação sanitária brasileira, nenhum produto de uso medicinal poderá ser industrializado, exposto à venda ou entregue ao consumo antes de registrado ^20^. Apesar da criação da categoria específica, esses produtos deveriam se adequar à regulamentação sanitária. Após os cinco anos estabelecidos como prazo, a partir da licença, os produtos autorizados para fabricação e comercialização deverão ser registrados junto à Anvisa. Até lá, a responsabilidade pelos riscos recai sobre fabricantes, prescritores e pacientes instados a assinar Termo de Consentimento Livre e Esclarecido (TCLE). Assim, percebe-se um contexto de possível assimetria de informação que pode causar maior ônus para os pacientes.

Mas o que falar sobre a quantidade de produtos autorizados a importação? Para esses, não há previsão de registro. Os quase 600 produtos nessa categoria continuarão se valendo dos requisitos regulatórios frágeis ou mesmo inexistentes, que hoje vigoram? Essa realidade privilegia aqueles que obtêm, por meio de recursos próprios ou via judicial, quaisquer produtos para quaisquer indicações. A situação da judicialização de produtos, fomentada pela pressão social, é tensionada pela relativa falta de evidências, e pela insegurança jurídica, do momento em que esses produtos não são medicamentos. A atuação da Comissão Nacional de Incorporação de Tecnologias no Sistema Único de Saúde (Conitec) é fundamental para direcionar a questão em prol da sustentabilidade do SUS.

A utilização terapêutica de um princípio ativo depende de comprovação de eficácia e de segurança para uma dada indicação. Os efeitos adversos da cannabis medicinal, tal qual as evidências de eficácia, ainda não apresentam resultados conclusivos. Há associação moderada para risco de eventos adversos com nabilona, nabiximol e CBD ^17^. Eventos cardiovasculares, psiquiátricos, gastrointestinais e sobre o sistema imune são relatados na literatura ^21^. Com produtos que contenham THC, o paciente poderá sofrer distúrbios relacionados tanto à concentração quanto ao uso crônico, como psicoses, ou assemelhados à dependência, sendo mais graves em pacientes suscetíveis ^19^ ^,^ ^21^ ^,^ ^22^ ^,^ ^23^.

Nos Estados Unidos, no debate sobre a regulação da cannabis medicinal, a questão da segurança vem sendo discutida no que tange à limitação de informações sobre efeitos adversos de produtos aprovados, indicando a necessidade da condução de estudos clínicos ^24^.

No contexto brasileiro, o monitoramento de eventos adversos demanda incentivo da Anvisa aos prescritores e pacientes, para notificação, contando com o alinhamento dos conselhos profissionais para proteção do paciente, zelando por indicações sustentadas por evidências.

## Quem molda a regulação?

Ainda que as contribuições da sociedade ajudem a moldar a regulação, as lacunas normativas abrem caminhos para um mercado nem sempre alinhado aos interesses sanitários. O resultado é o aumento progressivo do número de empresas buscando atuar nesse nicho, que se mostra lucrativo e permeável.

A Anvisa cria alternativas para permitir que os produtos contendo cannabis, que não são medicamentos e, portanto, que não foram avaliados quanto à eficácia, segurança ou qualidade, sejam acessados por pacientes munidos apenas de prescrição. Nesse caso, tão mais grave é o fornecimento, pelo SUS, de produtos que não atendam ou não se adequem aos requisitos sanitários previstos na legislação.

Espera-se que a Anvisa se posicione mais assertivamente no que tange à regulação da cannabis, tanto em relação aos usos indicados, de acordo com evidências disponíveis, como em relação à disponibilidade de produtos autorizados para importação e comercialização no país. A agência vem demonstrando, historicamente, determinação em proteger a população brasileira; os recentes exemplos vivenciados com a COVID-19 dão conta desse fato. Espera-se a mesma postura na regulação do mercado canábico, assim como de outros mercados relevantes que impactem a saúde da população.
